# Case Report: A rare transthyretin mutation p.D58Y in a Chinese case of transthyretin amyloid cardiomyopathy

**DOI:** 10.3389/fcvm.2024.1374241

**Published:** 2024-05-22

**Authors:** Jibin Lin, Jiangtong Peng, Bingjie Lv, Zheng Cao, Zhijian Chen

**Affiliations:** ^1^ Department of Cardiology, Union Hospital, Tongji Medical College, Huazhong University of Science and Technology, Wuhan, China; ^2^ Hubei Key Laboratory of Biological Targeted Therapy, Union Hospital, Tongji Medical College, Huazhong University of Science and Technology, Wuhan, China; ^3^ Hubei Provincial Engineering Research Center of Immunological Diagnosis and Therapy for Cardiovascular Diseases, Union Hospital, Tongji Medical College, Huazhong University of Science and Technology, Wuhan, China; ^4^ Department of Cardiology, Taihe Hospital, Hubei University of Medicine, Shiyan, Hubei, China

**Keywords:** transthyretin amyloid cardiomyopathy, amyloid, genetics, mutation, case report

## Abstract

Hereditary transthyretin amyloid (ATTRv) cardiomyopathy (CM) is caused by mutations in the TTR gene. TTR mutations contribute to TTR tetramer destabilization and dissociation, leading to excessive deposition of insoluble amyloid fibrils in the myocardium and finally resulting in cardiac dysfunction. In this article, we report a case of a Chinese patient with transthyretin mutation p.D58Y and provide detailed information on cardiac amyloidosis, including transthoracic echocardiography, cardiac magnetic resonance, and SPECT imaging for the first time. Our report aims to provide a better understanding of ATTR genotypes and phenotypes.

## Introduction

Amyloidosis is a heterogeneous group of clinical diseases characterized by the misfolding and deposition of amyloid proteins in different human tissues and organs, resulting in cell damage, tissue destruction, and organ dysfunction. In the case of cardiac amyloidosis (CA), nine amyloid fibril precursors have been reported to cause CA, including transthyretin (TTR) and immunoglobulin light chain, which account for more than 95% amyloid cardiomyopathy (1–3). The pathobiology of transthyretin amyloid includes TTR misfolding and aggregation, resulting in restrictive cardiomyopathy, heart failure, and other clinical symptoms (4–6). TTR is a 56 kDa homo-tetrameric protein composed of four 127-amino acid monomers. It is encoded by genes located on human chromosome 18q12.1. TTR is mainly transcribed in liver cells and can also be found in the brain, retina, and choroid plexus. Its primary role is to transport thyroxine- and retinol-binding proteins, with the stability of its structure closely associated with its function.

Transthyretin amyloidosis (ATTR) comprises wild-type (genetically normal, ATTRwt) and hereditary (substitution or deletion mutations, ATTRv) amyloidosis. In ATTR amyloidosis, the TTR protein instability is caused by genetic variations in ATTRv and age-related instability in ATTRwt. This instability leads to the disassociation of tetramers into monomers, which finally polymerize into insoluble fibrils. Based on a TTR mutation database (http://amyloidosismutations.com/mut-attr.php), more than TTR gene 140 variants were reported (7). Within these, the V30M (p.Val50Met, valine-to-methionine at amino acid position 50) mutation was by far the most studied one and believed to be the most common one worldwide (8). On the other hand, Val122Ile (p.Val142Ile) is the most common mutation responsible for familial amyloid cardiomyopathy. The clinical spectrums of ATTRv varied from exclusive neurological involvement, mix phenotype, to predominant cardiac involvement (9). In p.V50M, the extracardiac manifestations especially neurologic phenotypes were observed. Other mutations, such as p.V142I, p.T80A, and p.I88l**,** on the contrary, were reported to be predominantly cardiac phenotypes.

In clinical practice, ATTR-CA diagnosis is challenging. The red flags and caveats in CA should attract enough attention. In recent years, ATTR-CA has been increasingly diagnosed owing to improved clinical awareness and advances in diagnosis methods. In addition, the prevalence of ATTR-CA, especially ATTRwt, is believed to be much higher than previously thought (10, 11). Despite the advances in ATTR mechanisms, research, and therapeutic approaches, there is still a long way to go for ATTR treatment. For untreated ATTRwt patients, the median survival from diagnosis is 3.6–4.8 years; for ATTRv patients, 5.8 years; and for Val122Ile patients, only 2.6 years (2). Fortunately, there is now a TTR tetramer stabilizer, tafamidis, available for ATTR treatment. In addition, clinical trials involving TTR-specific antisense oligonucleotides (ASOs) and small interfering RNAs (siRNAs) are currently underway and are expected to be effective.

Here, we report a rare transthyretin mutation p.D58Y in a Chinese patient and provide detailed CA information for the first time. Our report aims to enhance our understanding of the genotypic and phenotypic spectra of ATTR hereditary.

## Case presentation

A previously healthy 55-year-old man underwent a physical examination where a reduction in ejection fraction (EF) was found accidentally. Subsequently, he was prescribed anticardiac remodeling drugs. Clinical characteristics are presented in Table 1. The patient suffered from syncope during climbing 10 days before admission. He had a history of hypertension for 8 years with the highest systolic blood pressure of 158 mmHg. Additionally, he underwent cholecystectomy 1 year prior and reported chronic diarrhea for several years without undergoing examination.

**Table 1:** Clinical characteristics summary of the patient.

Age, 55; sex, male; BMI, 21.72
NYHA functional class, III; 6 min walk test, 400 m
Serum biochemical parameter:
NT-ProBNP, 4,050.0 pg/ml; hsTNI, 83.6 ng/L; eGFR, 113.14 ml/min/(1.73 m^2^)
Echocardiographic parameters:
IVS (for inferior wall), 24 mm; LV (mm), 51; LA, 43; RA, 50; RV, 45
LVEF, 43%; RVFAC, 38%; E/A, >2

BMI, body mass index; NT-proBNP, N-terminal pro-B-type natriuretic peptide; hsTNI, high-sensitivity troponin I; eGFR, estimated glomerular filtration rate; IVS, interventricular septum; LV, left ventricular; LA, left atrium; RA, right ventricular; RA, right atrium; LVEF, left ventricular ejection fraction; RVFAC, right ventricular fractional area change; E/A, mitral valve E-wave/A-wave ratio.

**Figure 1:**
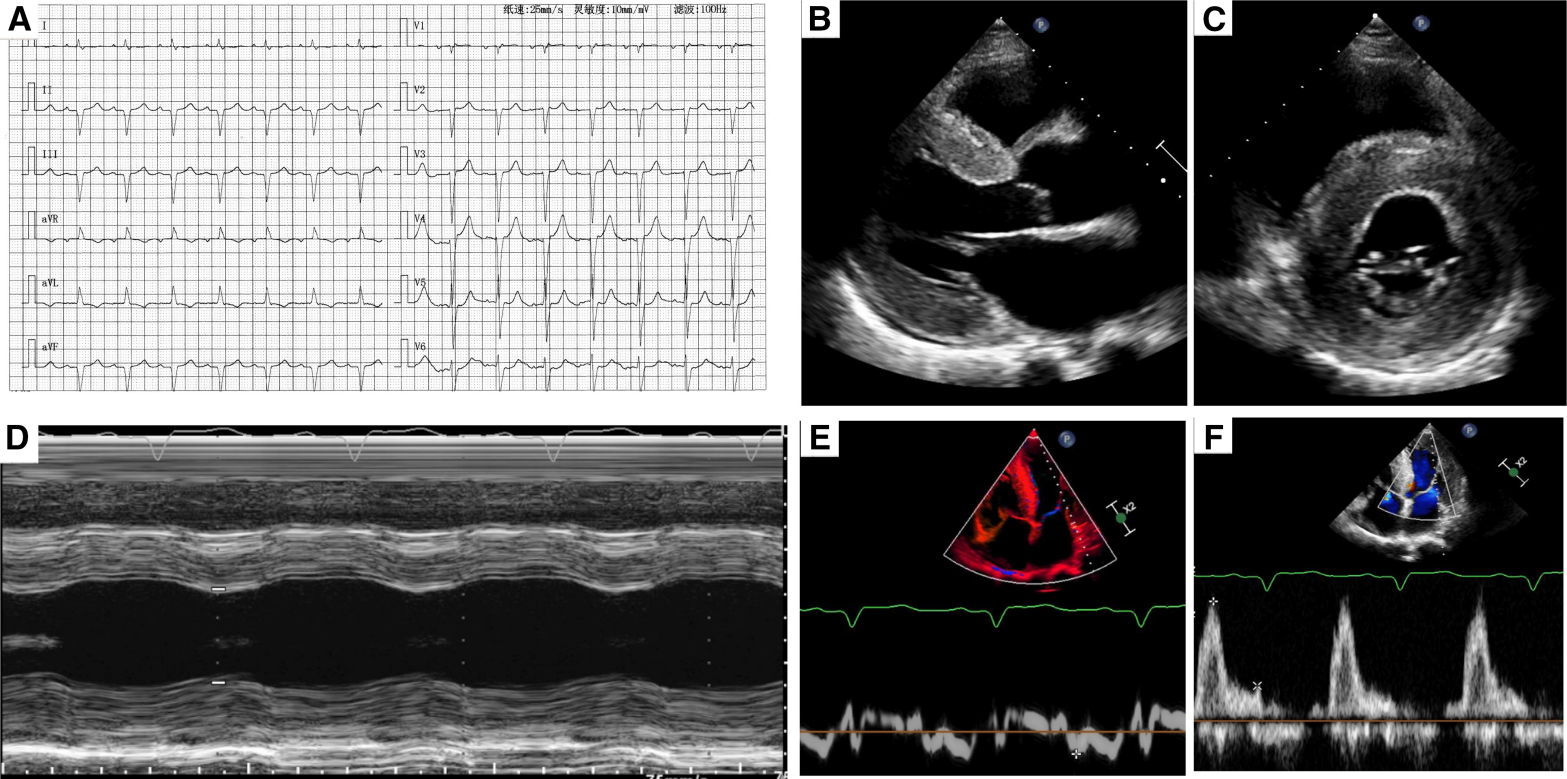
(**A**) A typical electrocardiogram. (**B,C**) Two-dimensional transthoracic echocardiography showed left ventricular hypertrophy and myocardium speckled pattern. (**D**) The contractility function was reduced. (**E,F**) Diastolic dysfunction (stage III) was found by pulse-waved Dopper echocardiogram.

ECG showed old inferior and interior wall myocardial infarction (Figure 1A). For laboratory testing, NT-proBNP was found significantly increased at 4,050.0 pg/ml (threshold value, 125 pg/ml). Complete blood count, myocardial enzymes, and biochemical examination were normal.

Transthoracic echocardiography demonstrated diffuse hypertrophy of the left ventricular wall and “ground-glass” enhancement of myocardial echogenicity. The thickness for basal, middle, and apical segments of the interventricular septum was 24, 22, and 21 mm, respectively. The corresponding thickness of the segments for the inferior wall was 24, 18, and 22 mm, respectively. In addition, the contractility function was reduced with an estimated LVEF of 43%. The right ventricular wall motion was normal with a right ventricular fractional area change of 38%. Furthermore, bi-atrial enlargement and diastolic dysfunction (stage III) were observed. What's more, mild to moderate insufficiency was found in the aortic, mitral, and tricuspid valves (Figures 1B–E).

Coronary arteriography showed coronary artery was normal. In addition, a dynamic electrocardiogram showed that the patient had no fast or chronic arrhythmia but only 625 ventricular premature beats in 24 h. The average heart rate was 62 bpm (min, 62; max, 93 bpm). What's more, craniocerebral MRI and MRA did not show significant abnormality.

**Figure 2:**
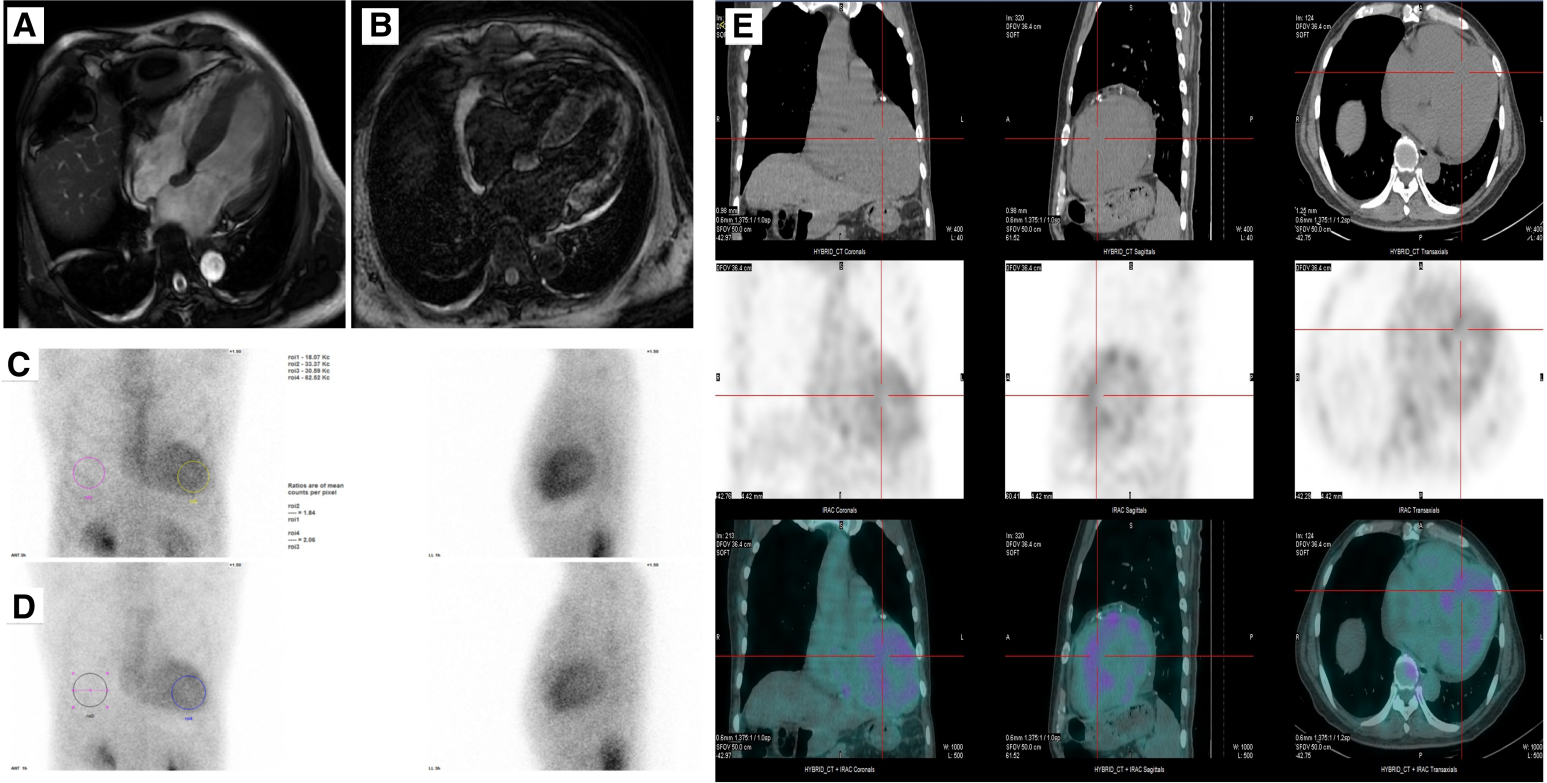
(**A,B**) Cine and late gadolinium-enhanced MRI. (**C–E**) A SPECT imaging using 99mTc-PYP scintigraphy showing uptake of 99mTc-PYP by cardiac at 3 h and 1 h.

Cardiac magnetic resonance was then used to evaluate cardiomyopathy due to his diffuse hypertrophy and reduced EF value. The first-pass perfusion imaging showed decreased subendocardial perfusion in the left ventricular walls. Delayed-enhancement magnetic resonance imaging revealed the left ventricle with diffuse granular enhancement, mainly under the endocardium (Figures 2A,B**)**. The patient had no family history of amyloidosis. Serum kappa, lambda light chains, and kappa/lambda free light chains ratio were normal, and no monoclonal protein was detected. Subsequently, a SPECT imaging using 99mTc-PYP scintigraphy was done. The result showed a significantly higher cardiac uptake than bone (SQA grade 3), indicating the possibility of transthyretin amyloid deposition (Figure 2C–E). A genetic analysis of the TTR was then performed, and the result revealed a rare mutation NM_000371.4 c.172G>T resulting in p.D58Y for the Chinese population (chr18:31592998-31592998, human genome 38).

According to the new British ATTRm prognostic staging scores, this patient was in stage II (NT-proBNP, >3,000 pg/ml), indicating a median survival time of approximately 46.7 months (12). The patient is currently undergoing treatment with tafamidis (20 mg p.o. qd) and is under regular follow-up.

## Discussion

 Here, we reported a rare transthyretin mutation p.D58Y(c.172G > T) in a Chinese patient. Due to historical reasons, there was inconsistency among different groups in the numbering of amino acids. Some labs number them according to the beginning of the mature protein, while others include the 20-amino acid signal sequences in numbering the unprocessed gene product. There have been some reports of the same amino acid mutation of the TTR gene, including p.D58A, p.D58V, and p.D58Y, in which the amino acid aspartic acid (D) was replaced by alanine (A), valine (V), and tyrosine (Y), respectively (Table 2).

**Table 2:** The basic clinical spectrums of ATTRv patients.

Patient no.	Mutation	Age at onset	Age at diagnosis	Gender	National/regional origin	Phenotype	First symptom	Ref.
	D38A (p.D58A)	\		\	\	\	\	(13)
1	p.D58Y (c.172G>T)	56	58	Female	Brazilian	Mixed	Paresthesias	(14)
2	p.D58Y (c.172G>T)	\	68	Female	Brazilian	Neurological	Leg weakness and chronic constipation	(14)
3	p.D58Y (c.172G>T)	\	71	Male	Caucasian	\	\	(15)
4	p.D58A (c.173A>C)	64	66	Male	Korean	Mixed	Diarrhea	(16)
5	p.D58A (c.173A>C)	58	63	Male	Korean	Mixed	Paraesthesia	(16)
6	p.D58A (c.173A>C)	63	65	Male	Korean	Mixed	Diarrhea	(16)
7	p.D58A (c.173A>C)	47	51	Male	Korean	Mixed	Diarrhea	(16)
8	p.D58 V(c.173A>T)	62	65	Female	Korean	Neurological	Paresthesia	(17)
9	p.D58A(c.173A>C)	44	48	Male	Korean	Mixed	Paresthesia, hand weakness	(17)
10	p.D58A(c.173A>C)	58	59	Male	Korean	Mixed	Paresthesia, hand weakness	(17)
11	p.D58A(c.173A>C)	57	58	Female	Korean	Mixed	Paresthesia	(17)
12	p.D58A(c.173A>C)	57	63	Male	Korean	Mixed	Paresthesia	(17)
13	p.D58A(c.173A>C)	64	66	Male	Korean	Mixed	Diarrhea	(17)
14	p.D58A(c.173A>C)	47	52	Male	Korean	Mixed	Diarrhea	(17)
15	p.D58A(c.173A>C)	56	62	Female	Korean	Mixed	Dyspnea	(17)
16	p.D58A(c.173A>C)	\	53	Male	Korean	Mixed	Dyspnea, dizziness	(18)
17	p.D58A(c.173A>C)	\	\	Male	Korean	\	\	(18)
18	p.D58A(c.173A>C)	78	82	Female	Japanese	Mixed	dysesthesia	(19)
19	p.D58A(c.173A>C)	48	57	Female	Japanese	Mixed	edema	(19)
20	p.D58A(c.173A>C)	78	80	Male	Japanese	Mixed	dysesthesia	(20)
21	p.D58A(c.173A>C)	60	63	Female	Japanese	Mixed	dizzy	(21)
22	p.D58 V(c.173A>T)	49	52	Male	Chinese	Mixed	Orthostatic hypotension	(22)
23	p.D58 V(c.173A>T)	51	56	Male	Poland	Mixed	Loss of weight, numbness and paresthesia	(23)
24	p.D58 V(c.173A>T)	\	71	Male	Spanish	Mixed	\	(24)
25	p.D58 V(c.173A>T)	\	65	Female	Spanish	Mixed	Dyspnea	(24)
26	p.D58 V(c.173A>T)	\	39	Female	Spanish	No	No	(24)
27	p.D58 V(c.173A>T)	58	\	\	Ghanaian	Mixed	\	(25)
28	p.D58 V(c.173A>T)	\	42	Female	African	\	\	(15)

According to the 14-year update Transthyretin Amyloidosis Outcomes Survey (THAOS), which included 5,894 ATTR patients, there were 36 p.D58A patients (0.6% of all ATTR patients) involved (13). Unfortunately, no details about these 36 patients were given. Lavigne et al. (14) reported two Brazilian sisters with p.D58Y mutation, who suffered from late-onset neuropathy associated with CA. The first patient suffered from hand paresthesias at age 56. After 2 years of disease progression, she developed postural hypotension and syncope episodes and was then found to have CA. However, no evidence of cardiac involvement was provided. The second patient suffered from a rapidly progressive leg weakness and chronic constipation. A severe sensory and motor axonal neuropathy was revealed by electromyography. It is worth noting that her cardiac function was normal. What's more, another two other groups reported p.D58Y mutants but again without details (15, 26, 27). Hence, in contrast to previous studies, we provided here detailed clinical phenotypes for ATTR p.D58Y mutation, especially the CA information for the first time.

Interestingly, after carefully searching, the other similar mutations for this amino acid aspartic acid were most reported in East Asia. Jang et al. (16) reported 4 p.D58A patients from seven mutations and indicated that p.D58A was the predominant TTR mutation in Korean patients. They found that Asp58Ala had older disease onset ages and higher gastrointestinal involvement incidence. Similarly, Choi et al. (17) reported the characteristics of 18 South Korean ATTR patients and found that p.D58A was the most common mutation pattern (8/18). These eight patients shared similar clinical symptoms, such as polyneuropathy and cardiac disease. Three of them complained of severe diarrhea with weight loss, which was consistent with our case. This patient had suffered from diarrhea for several years, and it cannot be ruled out whether diarrhea was associated with amyloidosis. In addition, Cho et al. (18) reported a p.D58A variant Korean male with clinical polyneuropathy presentation, whose amyloid was distributed in the heart and colonic mucosa. It seems that amyloid deposition distributed in the heart and colonic mucosa with polyneuropathy was p.D58A ATTR's clinical feature. Moreover, future researches are needed to clarify whether digestive tracts are involved in these ATTR D38 (aspartic acid at position 58) mutations. After fully communicating with the patient and his family in this case, there were no other somatic neuropathy or autonomic dysfunctions. However, it could be better if assessments such as electromyography and histopathological examination of the nerve were performed in future follow-ups with the patient's consent.

In addition, Yazak et al. (19) and Yazaki et al. (28). reported two Japanese female patients with p.D58A mutation. They found that these mutants suffered earlier cardiac dysfunction with cardiomegaly and heart failure than other mutants. They also found pulmonary parenchyma was diffusely amyloid deposition by autopsy in p.D58A patients. Moreover, another two labs, Tachibana and Kishikawa, also reported one Asp38Ala case each (20, 21). For Chinese data, He et al. (22) summarized the clinical characteristics and prognosis of 23 Chinese ATTR-CM patients, including p.D58V. They reported that the clinical presentations of this p.D58V patient were heart and nerve mix phenotype with orthostatic hypotension as the initial manifestation. In addition, they found that Asp38Val belonged to the early-onset group (<50 years old).

In addition, tmutations were also reported outside East Asia. Gillmore et al. (15) mentioned one African female with p.D58V mutation, but no details were given. Lipowska et al. (23) reported the genetic and clinical presentation of transthyretin-related familial amyloid polyneuropathy (ATTR-FAP) in Poland. They reported one 51-year-old Asp38Val (p.Asp58Val) patient with first manifestation: loss of weight, numbness, and paresthesia in feet. Augustin et al. (24) reported a large pedigree from a 71-year-old Spanish man with p.D58V patient who had predominant late-onset heart involvement with variable polyneuropathy. What's more, Lachmann et al. (25) reported one Ghanaian patient with p.D58V mutation, who presented with CA but the predominant clinical feature was neuropathy.

The worldwide presence of Asp mutation showed the importance of this amino acid in ATTR pathogenicity. As we know, the ATTR structure stability was closely associated with its function. The kinetic and thermodynamic stability studies would be very important for better understanding ATTR mutants. Recently, Ma et al. (29) characterized the kinetic and thermodynamic stabilities of a novel Chinese c.175G>T p.D59Y mutant, which was very close to the c.172G>T p.D58Y mutant in our study. They found that p.D59Y was less stable than WT TTR and tafamidis showed potential benefits to p.D59Y ATTRv. It was interesting that not all TTR variants were associated with amyloidosis. Using ligand binding and structural studies, Almeida et al. (30) found that the Met 119 variant would induce residue Leu-110 to move to a position closer to the hormone, resulting in increased T4 binding affinity. Rosen et al. (31) and Curtis et al. (32) also reported increased T4 affinity in Met 119 variant. What's more, in opposition to other TTR variants, Coelho et al. (33) found that Met 119 substitution may induce tetrameric structure stabilization and result in lower amyloidogenic potential. The effects of these D38 (aspartic acid at position 58) mutations on ATTR structure stability and pathophysiology is an important question and needs to be addressed in the future. Furthermore, the main limitations of this study include the lack of biopsy and pedigree and the short follow-up period.

## Conclusion

In this article, we presented a rare case of a Chinese patient with transthyretin mutation p.D58Y and provided detailed CA information for the first time. Presenting this p.D58Y ATTR case aims to provide a better understanding of ATTR genotypes and phenotypes.

## Data Availability

The original contributions presented in the study are included in the article/Supplementary Material, further inquiries can be directed to the corresponding author.
